# StructureDistiller: Structural relevance scoring identifies the most informative entries of a contact map

**DOI:** 10.1038/s41598-019-55047-4

**Published:** 2019-12-06

**Authors:** Sebastian Bittrich, Michael Schroeder, Dirk Labudde

**Affiliations:** 1 University of Applied Sciences Mittweida, Mittweida, 09648 Germany; 2 Biotechnology Center (BIOTEC), TU Dresden, Dresden, 01307 Germany; 3 Research Collaboratory for Structural Bioinformatics Protein Data Bank, University of California, San Diego, La Jolla, CA 92093 USA

**Keywords:** Protein folding, Protein folding, Protein structure predictions, Software

## Abstract

Protein folding and structure prediction are two sides of the same coin. Contact maps and the related techniques of constraint-based structure reconstruction can be considered as unifying aspects of both processes. We present the Structural Relevance (SR) score which quantifies the information content of individual contacts and residues in the context of the whole native structure. The physical process of protein folding is commonly characterized with spatial and temporal resolution: some residues are Early Folding while others are Highly Stable with respect to unfolding events. We employ the proposed SR score to demonstrate that folding initiation and structure stabilization are subprocesses realized by distinct sets of residues. The example of cytochrome c is used to demonstrate how StructureDistiller identifies the most important contacts needed for correct protein folding. This shows that entries of a contact map are not equally relevant for structural integrity. The proposed StructureDistiller algorithm identifies contacts with the highest information content; these entries convey unique constraints not captured by other contacts. Identification of the most informative contacts effectively doubles resilience toward contacts which are not observed in the native contact map. Furthermore, this knowledge increases reconstruction fidelity on sparse contact maps significantly by 0.4 Å.

## Introduction

**Figure 1:**
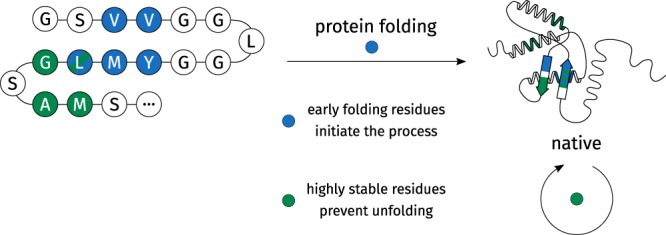
Studying protein folding by hydrogen-deuterium exchange. Most proteins adopt a native conformation autonomously in the process of protein folding^16,68^. A small number of Early Folding Residues (EFR, depicted in blue) initiate the folding process as their surroundings change before that of other residues^3^. Analogously, folded proteins can be analyzed with respect to their stability. Highly Stable Residues (HSR, depicted in green) comprise regions which are particularly resilient to unfolding events^6^.

Proteins are chains of amino acids which adopt complex, three-dimensional structures. This particular arrangement allows proteins to catalyze chemical reactions, transmit signals between cells, or recognize other molecules. The connection of protein sequence and structure is unclear and constitutes the protein folding problem. One promising technique to gain detailed insights into the process of protein folding (Fig. 1) are pulse-labeling hydrogen-deuterium exchange (HDX) experiments^1–3^. In the process of protein folding, a denatured protein chain adopts a native, functional conformation. HDX allows to study the process with spatial and temporal resolution and folding events of particular residues can be related to particular time steps. Early Folding Residues (EFR, blue in Fig. 1) initiate the formation of stable local structures starting from the denatured protein chain^1,2,4,5^. In contrast, Highly Stable Residues (HSR, green in Fig. 1) constitute regions in the native conformation^6^ which are resilient to unfolding events (e.g. as natural phenomenon^7^ or change in temperature or pH^8^). Both EFR and HSR are key aspects to understand the protein folding process^3,9^; standardized data is provided by the Start2Fold database^10^. The defined-pathway model was proposed based on these observations. It considers protein folding to be a deterministic process where defined regions initiate the folding process and fragments assemble stepwise to form the native conformation^2,11,12^ by establishing tertiary contacts^2,13–15^. EFR constitute the folding nucleus and seem to determine the order in which certain sequence fragment fold. However, the relevance of EFR on the structural integrity of a protein structure is little explored. One reason is that it is currently not possible to assess the role of a contact or residue regarding the structural integrity of a protein; especially an *in silico* approach suitable for large-scale studies is needed to assess the relevance of EFR and HSR. Closely related to the protein folding problem are protein design and the prediction of structures from sequence^16^.

**Figure 2:**
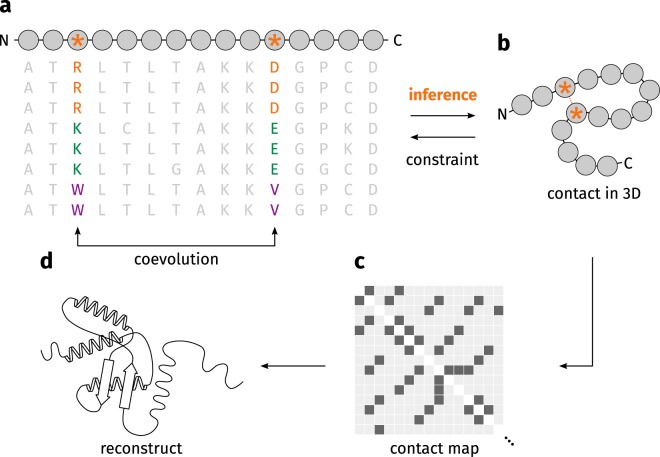
Protein structure prediction by coevolution techniques. (**a**) For a given sequence, homologous sequences can be used to create a multiple sequence alignment. Some positions coevolve (depicted by an orange asterisk): where for a change at one position a suitable change at the second position can be observed. (**b**) This connection at sequence level implies spatial proximity of both residues. (**c**) Coevolving residues can be represented by a contact map. (**d**) The predicted contacts are used as constraints of a subsequent structure reconstruction in order to find an optimal three-dimensional structure. Figure adapted from^17^.

Coevolution techniques^17–20^ propose an elegant approach to predict the structure of proteins from the abundance of sequences known today. For a given sequence, homologous sequences are retrieved and subsequently aligned via multiple sequence alignment (Fig. 2a). Therein, some residues at defined sequence positions are conserved while others may change freely. A small number of residues are coupled to other positions: when one position changes the coupled position will change accordingly. This constraint implies the spatial proximity of both residues: even if they are separated at sequence level, they show a signal of coevolution because they are in contact at structure level (Fig. 2b)^17^. The predicted contacts constitute a contact map (Fig. 2c) which can be used as set of constraints for a subsequent structure reconstruction (Fig. 2d). Conformations are sampled by a stochastic process in order to fulfill as many constraints as possible^21^.

A contact map comprises the set of gathered constraints. Contact maps are matrices encompassing all pairs of sequence positions and usually contain a binary annotation whether two residues are in contact or not^22,23^. They are used to design and train coevolution techniques and are also their output. Subsequently, these predicted contacts are used as constraints for reconstruction algorithms^21,24,25^ in order to find conformations which fulfill the maximum number of constraints. Thus, coevolution techniques are capable of *ab initio* structure predictions which is not feasible by e.g. homology modeling approaches^26^. Predicted contacts used as constraints have also been demonstrated to speed-up molecular dynamics simulations by allowing for faster convergence^27^.

The success of coevolution techniques continues to revolutionize structural biology^18,28^ and spawned a comprehensive ecosystem of related methods revolving around contact maps. The recent iteration of the CASP experiment emphasizes the coming of age of contact prediction and the improvement of *ab initio* protein folding protocols^29–31^. Dedicated methods for the visualization and interpretation of contact maps were created^32–34^. Quality assessment of the predicted contacts becomes increasingly important as well. False positive predictions (i.e. non-native contacts not observed in the native structure of a protein) are common. They have detrimental effects on the usefulness of contact maps^22,23^. Peculiarly, such false positive predictions are difficult to spot^17^ and in reconstruction they impair the feasibility of all other contacts^35^. Thus, dedicated methods were designed to validate contact maps^34,36^. Other studies^23^ tried to elucidate the optimal contact definition by assessing its influence on reconstruction performance. Commonly, contacts stabilizing secondary structure elements (i.e. residues separated by less than six positions on sequence level) are ignored in the context of contact maps^37^. The range of the remaining contacts are considered short-range (sequence separation of 6–11), medium-range (12–23), or long-range (>23)^38^.

Contact maps do not only contain the information needed for protein structure prediction, but they also are potential tools to describe the fundamentals of protein folding. In 2007, Chen *et al*.^39^ pioneered the search for the most relevant contacts of a contact map and wanted to determine the minimal set of contacts which captures the fold of a protein. Therefore, they represented proteins by contact maps and selected random subsets with varying coverage. These subsets were then used as constraints in a structure reconstruction algorithm, the result was aligned to the native structure, and its fidelity was assessed by the root-mean square deviation (RMSD). As the number of constraints increased (i.e. more contacts of the native contact map are considered), the RMSD decreased because the reconstructs resembled the native structure increasingly well. A reconstruction is considered successful when the RMSD to the native structure is below a certain threshold and likely to resemble the correct fold^17,22,39,40^: in our study, we use the threshold of 4.0 Å by Marks *et al*.^17^. Good reconstructions have been shown to depend on a delicate balance of sequentially neighbored and sequentially separated contacts^39^. Sathyapriya *et al*.^40^ extended the study of Chen *et al*. and coined the term *structural essence* for the minimal set of fold defining contacts. They demonstrated that 8% of all contacts allow for the reconstruction of the correct fold of a protein because most information in a contact map is redundant. Furthermore, a rational selection of contacts can outperform a random selection of equally many contacts with respect to reconstruction quality. However, such a configuration is difficult to compose^40^. Duarte *et al*. showed that consideration of all contacts leads to reconstruction qualities around 2 Å^23^.

The annotation of EFR and HSR provided by the Start2Fold database^10^ is valuable information to understand the protein folding problem and has also implications for the prediction of protein structures. Contact maps are the cornerstone of contemporary structure prediction methods. The surrounding ecosystem of reconstruction algorithms may elucidate the protein folding process by pinpointing the most important contacts for structural integrity. Additionally, the relevance of EFR and HSR in the context of protein structure prediction provides qualitative insights. Several studies identified a small number of key residues for the *in vitro* folding process. It has also been shown that the information content of experimentally determined NMR restraints varies drastically^41^. Is the same true for *in silico* folding: do some contacts convey more structural information than others? For a long time, *in silico* folding simulations improved the understanding of the protein folding process^42,43^, potentially contact maps provide an even more tangible connection of both aspects. To address these questions, we propose the Structural Relevance (SR) score which quantifies the amount of information an individual contact or residue provides for an *in silico* reconstruction process.

## Results

A subset of proteins from the Start2Fold database^10^ was analyzed. The folding and stability characteristics of the corresponding proteins have been determined by HDX experiments^8,10,44^ and these properties may relate to the most relevant contacts of a contact map and constitute a direct connection of protein folding *in vitro* and structure prediction *in silico*. We only considered entries for which both EFR and HSR were annotated, totalling in 30 proteins. For this dataset of proteins with known *in vitro* folding characteristics, we aimed to identify the most informative contacts *in silico* by extending previous studies^22,39–41^ and assess if our findings correlate with the experimentally determined folding characteristics.

**Figure 3:**
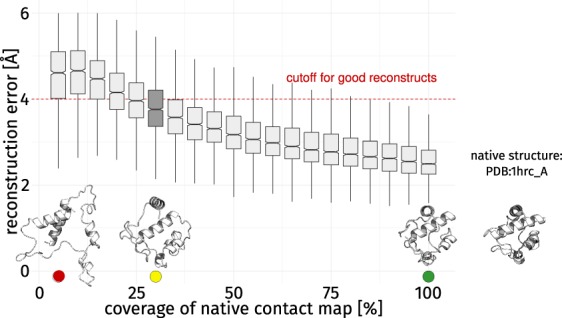
Reconstruction error by percentage of contacts. When more contacts are considered, the average reconstruction error decreases^39^ and the same is true for the variance of each bin. For the assessment of the SR of contacts, 30% of all native contacts (box plot filled dark gray) were chosen as compromise because it ensures reconstructs of average quality while the corresponding contact maps are still sensitive to the removal or addition of individual contacts (as indicated by a big shift in reconstruction error with respect to the neighboring bins). Renderings of four structures are provided to make the influence of the coverage of the native contact map more tangible. They resemble knowledge of 5%, 30%, and 100% of all native contacts as well as the native structure (PDB:1hrc_A, isolated on the right).

Currently, no strategy exists to quantify the information provided by a single contact. We argue that constraint-based reconstruction algorithms such as CONFOLD^21^ can access this information when employed in a modified setting. Using structures deposited in the PDB archive^45^, native contact map representations of all proteins were computed (consult the method section for details). All-atom models can be reconstructed from these reduced representations using CONFOLD^21^. The fidelity of these reconstructs was assessed by a structure alignment^46^ to the native structure and is considered the reconstruction error^39,40^. The average RMSD of these reconstructions approaches 2 Å (Fig. 3), as described in literature^40^. Detailed identifiers and results are given in Supplementary Fig. 1 which also shows that no successful reconstructions could be achieved for Start2Fold entry STF0009 (PDB:1a64_A). In general, knowledge of 100% of entries in the native contact map leads to good reconstructs which resemble the native structure (Fig. 3). Structural constraints are redundant^41^ and, thus, it is not possible to directly assess the information conveyed by a single contact^39–41^. Therefore, we decreased the coverage of the native contact map in 5% steps which leads to an increase in reconstruction error. Sparse contact maps using a random selection of 5% of all contacts do not yield good reconstructs below the threshold of 4 Å for which the reconstruct would successfully resemble the native fold. The reconstruction process using more contacts becomes more robust as the distributions decrease in variance. Generally speaking, there is a sweet spot at 30% coverage where the yielded reconstructs resemble the fold of the native structure and are also sensitive to the removal or addition of individual contacts.

The idea of the StructureDistiller algorithm is to exploit the sweet spot at 30% coverage (Fig. 3) to quantify the information provided by a single contact. The reconstruction error for the so-called baseline reconstructs with a coverage of 30% can be determined. A contact is removed from the selection if it is present in the random selection of 30% of all contacts (and added otherwise) and so-called toggle reconstructs are computed for this slightly changed contact maps. Similar to the approach of Nabuurs *et al*.^41^, the information content of a contact is the decrease in RMSD which knowledge of a particular contact provides in relation to the absence of that contact. Supplementary Figure 1 shows that the optimal coverage is structure-specific and the determined default value of 30% is not generally the best choice, as also discussed in literature^17^. It would be possible to address this issue by determining the structure-specific value (e.g. sample a range of numbers and determine when reconstructs with RMSD below 4.0 Å or TM-scores above 0.5 are achieved) and using this value for all subsequent calculations. In summary, the StructureDistiller algorithm quantifies the Structural Relevance (SR) of a contact by disentangling it from other contacts mandatory for a meaningful reconstruction in the first place (details are described in the method section).

### The structural relevance of individual contacts and residues

We computed the SR score for all 5,173 contacts in the dataset by the presented StructureDistiller algorithm. The outputted score captures the average performance increase in Å, when a particular contact is considered for the reconstruction process compared to a reconstruction without knowledge of this contact (ΔRMSD). Positive SR scores indicate contacts which favorably contribute to reconstruction fidelity, whereas negative scores indicate native contacts which hinder or at least not substantially improve the process. The removal of an individual contact results in (negative) change in SR by 0.012 ± 0.253 Å (throughout the manuscript the standard error is given). In contrast, the addition of a contact leads to an increase by 0.022 ± 0.253 Å. Most contacts contribute positively to reconstruction performance. Only a small number of contacts is of high SR with similar tendencies shown by studies on contact maps^39,40^, NMR restraints^41^, and protein folding in general^47^. Correctly folded protein structures depend on a small number of key contacts. The high variance of the SR scores is the result of both the contact map sampling as well as the reconstruction routine^21^ being stochastic processes. Both operations are performed with ten-fold redundancy to limit this issue. The presented SR scores are the average values over all redundant runs.

We used several features (Table 1) to describe contacts in the dataset in more detail and assess their relation to the SR score. First, residue contacts are distinguished according to their sequence separation^38^. Short-range contacts (6–11) exhibit a significant decrease in the SR score. In contrast, long-range contacts (>23) of sequentially highly separated residues are more common and feature increased SR scores. The change is insignificant for medium-range contacts. Previously it has been shown that contacts within as well as between secondary structure elements are required for optimal reconstruction performance^39,40^. Commonly, reconstructions only consider residue pairs at least six positions apart at sequence level^38^, though there are cases where the usually ignored contacts may contribute valuable information pertaining the structure of loops^48^.

**Table 1:** Contact-level features influencing the SR (ΔRMSD) score.

feature	present	*n*	*μ*_SR_ [pm]	*σ*_SR_ [pm]	trend	*p*-value
short-range contact (6–11)	yes	1,120	1.4	8.1	↓	0.025
	no	4,053	2.2	8.6		
medium-range contact (12–23)	yes	1,271	1.8	8.6	—	0.161
	no	3,902	2.1	8.5		
long-range contact (>23)	yes	2,782	2.4	8.6	↑	0.002
	no	2,391	1.6	8.4		
hydrogen bond	yes	563	1.1	8.3	↓	0.018
	no	4,610	2.1	8.5		
hydrophobic interaction	yes	541	2.9	9.5	↑	<0.001
	no	4,632	1.9	8.4		
evolutionarily coupling	yes	1,461	2.2	8.4	—	0.203
	no	3,246	1.8	8.7		
top-scoring coupling	yes	1,020	2.4	8.3	—	0.059
	no	3,687	1.8	8.7		

Contact length refers to the sequence separation of the contact^38^. Hydrogen bond and hydrophobic interaction refers to contacts for which the respective interaction type was observed^49^. Evolutionary couplings by direct coupling analysis^17,51^, for some proteins no data could be computed. Top-scoring couplings are the first 0.4*L* contacts sorted by their coupling rank. *n* describes the number of observations, *μ* the corresponding average, and *σ* the respective standard deviation. The trend is given, i.e. does presence of this feature decrease (↓) or increase (↑) the SR scores. Insignificant change is represented by a dash (–).

Furthermore, we investigated the SR scores of non-covalent interactions such as hydrogen bonds and hydrophobic interactions. The PLIP algorithm^49^ was employed to detect non-covalent interactions, the tool also reports which atoms interact. A significant change in SR can be observed when a non-covalent interaction was detected between both partners of a contact. Hydrogen bonds exhibit lowered SR scores, whereas an increase can be observed for hydrophobic interactions. Hydrogen bonds primarily occur between backbone atoms of amino acids where they define and stabilize interactions between secondary structure elements. Some amino acids such as serine or threonine feature polar side chains which allow them to engage more flexibly in this type of non-covalent interaction. The importance of hydrogen bonds furnished by side chains for protein folding and stability has been shown^16,50^. Hydrogen bonds may feature lower SR scores because of their propensity to occur between polar amino acids at positions exposed to the solvent. In contrast, hydrophobic interactions primarily occur in the buried hydrophobic core of a protein where they are surrounded by many other residues which reduces the degree of freedom. Especially, the importance of tertiary contacts furnished by hydrophobic interactions has been shown^5,14^. Such interactions provide information on the correct assembly of distant parts of the protein and, thus, are relevant for structural integrity both during protein folding and for structure prediction.

Potentially the contact prediction method EVfold^17,51^ captures contacts with high SR scores and ignores those carrying little information. However, we do not observe a significant association with the SR score. Yet, a slight increase in SR can be observed, when two positions are evolutionarily coupled. A selection of the 0.4*L* top-scoring contacts (*L* refers to the sequence length) results in a more substantial, though still insignificant, change in SR. Many predicted couplings are not actually present in the native contact map due to the strict distance cutoff. Also, potential false positive predictions by the direct coupling analysis are not evaluated, which can be expected to have a negative effect on reconstruction quality^22^.

All previous results consider the SR scores of individual contacts. Properties of individual residues can be analyzed with the same reasoning (Table 2) by summing up the SR scores of all the contacts they participate in. Residues in loop regions have significantly lower SR than those in *α*-helices and *β*-strands. For secondary structure elements, backbone angles and hydrogen bonding patterns are used as additional constraints during reconstruction^21^ which may explain an overall performance increase. The previous association of hydrophobic interactions and SR score may be explained by a bias for buried residues; however, no significant association is observed at residue level. The annotation of EFR does not influence SR scores significantly, while the opposite is true for HSR (see below). Functional residues may not be of high SR, because binding sites tend to be exposed to the solvent and commonly have unfavorable conformations^52^. Residues for which evolutionary couplings are predicted by EVfold^17,51^ do not exhibit increased SR. This is probably because couplings are distributed uniformly and at least one coupling is present for most residues. However, filtering for the 0.4*L* top-scoring positions (i.e. regarding their cumulative coupling strength) does not lead to a significant change either.

**Table 2:** Residue-level features influencing the average SR (ΔRMSD) score.

feature	present	*n*	*μ*_SR_ [pm]	*σ*_SR_ [pm]	trend	*p*-value
early folding	yes	414	2.6	6.1	—	0.543
	no	2,115	2.5	6.2		
highly stable	yes	688	3.0	6.2	↑	<0.001
	no	1,731	2.1	6.1		
functional	yes	119	2.8	6.2	—	0.919
	no	2,078	2.6	5.9		
coil	yes	996	1.9	6.4	↓	<0.001
	no	1,533	2.9	6.0		
buried	yes	1,105	2.7	5.5	—	0.075
	no	1,424	2.4	6.7		
evolutionarily coupled	yes	1,975	2.6	6.2	—	0.754
	no	503	2.6	6.4		
top-scoring coupled	yes	1,117	2.6	5.7	—	0.492
	no	1,361	2.5	6.6		

Residues in coil regions and residues buried according to their relative accessible surface area were evaluated. Residues were assessed regarding their early folding and highly stable characteristics^10^. Annotation of functional residues from UniProt^69^. Considers evolutionary couplings and the 0.4*L* top-scoring positions according to the cumulative coupling strength^17,51^. *n* describes the number of observations, *μ* the corresponding average SR, and *σ* the respective standard deviation. The trend is given, i.e. does presence of this feature decrease (↓) or increase (↑) the SR scores. Insignificant change is represented by a dash (–).

### Analysis of early folding and highly stable residues

A direct connection to particular folding and stability characteristics is provided by the annotation of EFR which initiate and guide the folding process. However, according to the SR score we observe no change for EFR (Table 2). Contacts of HSR exhibit a significant increase in SR compared to unstable contacts. It is remarkable that contacts of EFR show no increase in SR despite their presumed role for the protein folding process^4,44^. A possible interpretation is that EFR primarily define stable, local structures^4,44^ due to their occurrence in sequence regions associated to high backbone rigidity. They form defined sequence regions with fewer possible backbone conformations and produce pivotal secondary structure elements. Therefore, EFR define the folding nucleus of a protein and sequentially encode the ordered secondary structure elements formed first. However the obtained SR scores suggest that crucial contacts between these secondary structure elements may be mediated by other residues which are not necessarily EFR themselves, but may occur in secondary structure elements containing EFR^1^.

Another aspect of the experimental data by Pancsa *et al*. is the annotation of residues which are strongly protected in stability measurements^10^. Such residues occur in ordered secondary structure elements and their contacts are beneficial to reconstruction performance. Rather than initiating the formation of the native structure (like EFR), HSR seem to manifest the native conformation. The differences in SR scores between EFR and HSR imply that two distinct process are realized by these two distinct sets of residues.

The defined-pathway model^2,11,12^ describes protein folding as a deterministic, hierarchic process. EFR occur in regions which autonomously fold first relative to the rest of a protein. Furthermore, this tendency does not depend on tertiary contacts in a protein structure, but is rather the direct consequence of the local sequence composition^1,10,44^. These stable, local structures may be secondary structure elements^3^ or larger autonomously folding units also referred to as foldons^2^. In a stepwise process, such local structures will subsequently establish tertiary contacts and assemble the native conformation of a protein^2,15,53^. The employed reconstruction method directly considers secondary structure elements, which are used to derive additional constraints. Therefore, most secondary structure elements should be represented successfully which may explain why we observe long-range contacts to be particularly important for structural integrity. It is also reasonable that the SR score of a contact increases with the distance at the sequence level: potentially, such constraints do not only enforce the correct placement of both residues but also have an indirect positive impact on the correct conformation of all residues in between.

The dataset of EFR and HSR^10^ provides valuable information to converge on the protein folding problem^3,9^. The Start2Fold dataset^10^ enables the direct connection of protein folding and structure prediction which is furnished by contact map representations. It is implied that EFR may initiate protein folding and determine the order in which local structures are assembled^2,12^ but they are of average relevance in terms of the SR score. HSR may not fold early but constitute regions of a protein which prevent spontaneous unfolding. Interestingly, regions of HSR are of high relevance for the formation and stabilization of the correct protein fold. David Baker^54^ showed that short-range contacts lead to fast folding whereas a high ratio of long-range contacts leads to a slow down. EFR initiate the folding process by establishing contacts to neighbors at the sequence level^2,44^. Furthermore, hydrophobic interactions, contacts of ordered secondary structure elements, as well as long-range contacts promote structural integrity. In a previous study^5^, we showed that EFR occur in ordered secondary structures and are embedded in a network of hydrophobic interactions. This implies that EFR may initiate the formation of local structures which can then assemble to actually stabilize the global structure of a protein by HSR.

### Disruption to cytochrome c induces molten globule state

**Figure 4:**
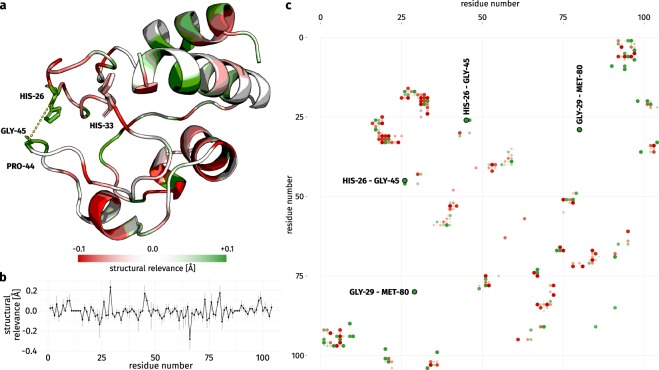
Cytochrome c (PDB:1hrc_A) colored by Structural Relevance (ΔRMSD). (**a**) Residues with high SR scores are depicted in green, those with negative SR are rendered in red. For gray residues no contacts were observed and no SR scores are reported. Disruptions to the hydrogen bond between HIS-26 and PRO-44 will induce a molten globule state when the association between both Ω-loops is lost^55,56^. StructureDistiller reports high SR for HIS-26, GLY-45, and the contact both share (yellow dashed line), though no direct contact is detected between HIS-26 and PRO-44 due to strict distance threshold of the employed contact definition. HIS-33 has been described as variable position lacking any structurally relevant contacts^55^ and this observation is manifested in the low SR score of this residue. The N- and C-terminal helices have been shown to initiate folding^8^ and exhibit high SR, especially for residues which constitute their interface. Other parts of the structure are primarily composed by coil regions, fixate a heme ligand, and show low SR. (**b**) Per residue SR as line chart. The standard deviation is given for each point. Residues without contacts exhibit a relevance of 0 Å. (**c**) Heatmap of the computed SR scores. Contacts of low relevance tend to be clustered together with high relevance contacts. The contact between GLY-29 and MET-80 has the largest SR score.

Ground truth on the structural importance of individual contacts is difficult to find – we used the dataset entry for cytochrome c as a case study. Cytochrome c (Fig. 4) contains two Ω-loops which are stabilized by a hydrogen bond between HIS-26 and PRO-44. The importance of this contact has been shown as disruptions induce a molten globule state^55,56^. Particularized folding studies^8^ have also identified the N- and C-terminal helices as foldons, i.e. autonomously folding units which initiate and guide the folding process. Besides that, wide parts of the structure are constituted of coil regions and fixate a heme ligand, thus potentially exhibiting increased structural flexibility.

The SR score computed by StructureDistiller of many residues of cytochrome c is neutral or even negative. Especially coil regions feature contacts which tend to decrease reconstruction fidelity. Remarkable are the high SR scores of HIS-26 and GLY-45 as well as their direct contact for which the score amounts to 0.172 Å (making it the fifth most relevant contact). No SR is reported for PRO-44 as it does not participate in any contacts according to the employed contact definition, though both groups are positioned in a way which would allow them to form a hydrogen bond. In literature^55^, the contact between HIS-26 and PRO-44 is reported as crucial for the correct conformation of cytochrome c. Disruptions will result in a loss of structure^55^, though the relevance of PRO-44 may also be attributed to the backbone rigidity introduced by the proline residue. The detection of relevant contacts and positions is fuzzy^57^, but the high scoring contact between HIS-26 and GLY-45 implies the importance of a contact between both Ω-loops for successful protein folding as well as structure reconstruction. Between GLY-29 and MET-80 the most relevant contact (with the highest SR) is located, it increases reconstruction fidelity by 0.563 Å on average. Furthermore, this contact is unique (i.e. no combination of contacts provides distance constraints transitively) and isolated from all other contacts in the map (Fig. 4c). This contact also occurs between two unordered coil regions, which implies that this structural information capturing the correct arrangement of these unordered protein parts is crucial for a successful reconstruction. Mutations to HIS-33 have been demonstrated to show no effect^55^ which is also captured by slightly negative SR score of −0.015 Å. Both N- and C-terminal helix contain residues with high relevance, especially in regions where both helices interact. The importance of these helix contacts has been shown previously^58^. The role of both helices as foldons^8^ points to high intrinsic stability. The SR score successfully spots contacts and residues crucial for structure integrity as shown in experiments^8,55,58^. The previously described contact between HIS-26 and PRO-44^55^ is absent as the result of a too strict contact definition, yet the necessity of structural information in this region is captured nevertheless.

### Knowledge of the most relevant contacts can increase reconstruction performance

**Figure 5:**
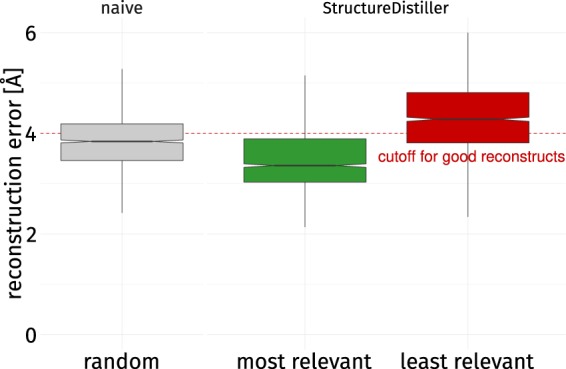
Impact on reconstruction performance by strategy. Three strategies were used to reconstruct structures of the dataset using a number of constraints equal to 30% of contacts in the native map. A random selection of contacts (gray), the most relevant ones by SR score (green), and the least relevant ones (red). The most relevant contacts yield the lowest reconstruction error when combined. This configuration outperforms a random selection of contacts significantly (*p*-value: <0.001). Previous studies^39,40^ have shown the difficulties in finding combinations of contacts yielding better reconstructs than a random selection. Using the least relevant contacts results in an increased error compared to the random selection (*p*-value: <0.001). When only a subset of all entries of a contact map can be considered (as it is commonly the case^34^ and reasonable for efficiency^40^), the subset of contacts chosen is crucial for reconstruction performance. This also shows that some contacts convey more information than others, as previously shown for NMR restraints^41^.

The subset of contacts with high SR scores should lead to good reconstructs when combined. To test this hypothesis, proteins were reconstructed using various subset selection strategies equal to 30% of all native contacts (Fig. 5). A baseline is obtained by selecting 30% of the contacts randomly (gray). Rational selections are based on sorting all contacts in a protein by its SR scores. The 30% top-scoring contacts represent the most relevant contacts (green). The bottom 30% represent the least relevant contacts (red). Other interesting aspects are contact distance and type: therefore short-range (6–11), long-range (>23) contacts, hydrogen bonds, and hydrophobic interactions were assessed (Supplementary Fig. 2).

The RMSD is used to quantify the fidelity of a reconstruct by aligning it to the native structure – high reconstruction errors occur for bad reconstructs. A random selection of 30% of contacts achieves 3.839 ± 0.599 Å. A combination of contacts by the most relevant strategy significantly outperforms the random strategy with an average reconstruction error of 3.479 ± 0.625 Å. Consideration of the least relevant contacts results in an increase in reconstruction error to 4.311 ± 0.687 Å.

Chen *et al*. assumed that no rational selection of contacts can surpass a random selection in terms of reconstruction fidelity^39^. Later, Sathyapriya and coworkers^40^ provided an algorithm capable of doing just that. It is especially remarkable that their approach merely evaluates which neighborhood is shared by a pair of residues. The main aspect of their algorithm is the selection of non-redundant contacts which can provide the maximum amount of information for a reconstruction when combined. Nabuurs *et al*.^41^ demonstrated the possibility to identify unique NMR restraints by a information-theory based approach. The selection of the most relevant contacts as determined by StructureDistiller constitutes a different approach to compose a set of contacts which allow for better reconstructs than a random selection. Of all native contacts two selections can be readily made. One is significantly better suited for reconstruction purposes than a random selection and whereas the other one performs significantly worse. It is also remarkable that a combination of long-range contacts performs significantly worse than the negated selection (Supplementary Fig. 2), despite individual long-range contacts exhibiting high SR scores (Table 1). This emphasizes the context-specificity of individual contacts^39–41^ and substantiates previous findings^39^, wherein both short- and long-range contacts are needed for good reconstructions.

### Increased resilience to non-native contacts

**Figure 6:**
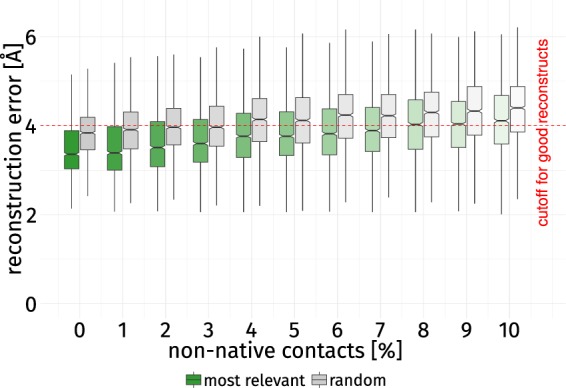
Influence of non-native contacts. The reconstruction error is given of for 30% of all contacts in the most relevant (green) and random (gray) bins with an increasing fraction of non-native contacts. In all cases, the most relevant contacts perform significantly better than a random selection when it comes to compensating non-native contacts (*p*-value < 0.001). E.g., the median performance of a random selection without non-native contacts is comparable to that of the best selection with 6% non-native contacts. When more than 3% non-native contacts are introduced into the random selection, the error of the majority of reconstructions lies above 4 Å, whereas the best selection can compensate more than double the number of non-native contacts before surpassing this threshold. Knowledge of the most relevant contacts as quantified by the StructureDistiller algorithm increases the resilience to non-native contacts as well as the overall reconstruction performance.

The sensitivity of a contact map to non-native contacts has been discussed before – even a small number of contacts not present in the native structure is detrimental to reconstruction performance^22^. As shown in the previous section, contacts with high SR allow for better reconstructs when sparse native contact maps are considered. Interestingly, the selection of the 30% most relevant contacts also can compensate the moderate introduction of non-native contacts (Fig. 6). This selection performs significantly better than a random selection in all considered cases. The introduction of non-native contacts quickly leads to reconstructions with errors above 4 Å as larger fractions of non-native contacts dilute the information captured by native contacts. When more than 7% non-native contacts are introduced to the most relevant selection, the majority of reconstructions is of bad quality. When 30% of all contacts are selected randomly, only 3% non-native contacts can be introduced before the error exceeds the threshold of 4 Å. The consideration of the most relevant contacts buffers the negative influence of non-native contacts (Table 3): median performance is comparable between reconstructions based on a random selection without non-native contacts and the selection of the best contacts diluted by 6% of non-native contacts.

**Table 3:** Reconstruction error introduced by non-native contacts.

non-native contacts [%]	*μ*_best_	${\tilde{{\boldsymbol{x}}}}_{{\bf{b}}{\bf{e}}{\bf{s}}{\bf{t}}}$	*μ*_random_	${\tilde{{\boldsymbol{x}}}}_{{\bf{r}}{\bf{a}}{\bf{n}}{\bf{d}}{\bf{o}}{\bf{m}}}$
0	3.479	3.360	3.839	3.840
1	3.498	3.390	3.903	3.910
2	3.598	3.510	3.971	3.965
3	3.665	3.600	3.996	3.965
4	3.808	3.765	4.135	4.140
5	3.840	3.765	4.117	4.120
6	3.882	3.820	4.211	4.240
7	3.931	3.890	4.229	4.225
8	4.028	4.030	4.256	4.300
9	4.038	4.040	4.292	4.330
10	4.140	4.110	4.354	4.400

For increasing rates of non-native contacts the reconstruction performance using 30% of the native contacts are given. *μ*_best_ refers to the average performance using the most relevant contacts, *μ*_random_ to that using a random selection of contacts. $\tilde{x}$ describes the median of the corresponding population. In all cases, the performance of the best bin is significantly better than that of a random selection.

Since even those native contacts can hinder reconstruction (as indicated by negative SR scores), it becomes evident that the correct ranking of contacts^21,36^ has a serious influence on reconstruction quality when subsets of contacts are considered. This knowledge also has implications for the design and training of contact prediction techniques. The insignificant association of evolutionary couplings and SR scores suggests that the most relevant contacts may not be easy to predict but can contribute significantly more information needed for the successful reconstruction of a protein.

Coevolution or supervised machine learning techniques are the basis for the prediction of contact maps^20,28,51^. Conventionally, contact predictors are designed and trained on collections of all native contacts in a dataset. Subsequently, the most reliable contacts are selected from all predictions; the size of this subset depends on sequence length^34^. This study shows that these subsets drastically change in meaningfulness as indicated by reconstruction fidelity. An implication is that it is not the optimal strategy to consider a random subset of contacts; reconstruction fidelity and information content per contact could increase when the contacts with the highest SR scores are considered. This would decrease the number of predicted contacts but may increase the reliability of their prediction by avoiding both false positive predictions and emphasizing contacts which promise to improve reconstruction fidelity the most while ignoring those which contribute only marginally. StructureDistiller enables this fine-grained interrogation of contact maps for the first time.

## Discussion

Contact maps are one of the most prominent tools in today’s structural bioinformatics^18,28^, though mere knowledge of residue contacts can neither describe all events of the protein folding process^59^ nor is it the optimal basis of structure prediction techniques^37^. Our study demonstrates that native contacts in a protein structure are not of equal importance for the reconstruction of the tertiary structure from this reduced representation. Similar observations have been made for NMR constraints^41^. StructureDistiller allows a more fine-grained analysis of contact maps and may pinpoint properties of contacts which can be associated with high Structural Relevance. Contacts of high Structural Relevance tend to be unique contacts for which no redundant backup exists as it is the case for the contact between two Ω-loops in cytochrome c^55^. The importance of this contact for the structural integrity also implies that high Structural Relevance scores may capture crucial positions for structure stability as shown by Highly Stable Residues.

The proposed strategy depends on some crucial assumptions and provides a number of points open for investigation in further studies. Residues in a protein are covalently bound and constraints on a residue will also affect neighboring residues. Thus, residue-specific information as complex as the Structural Relevance score should not be considered the absolute truth^57^. One of the most delicate aspects when handling contact maps is the used contact definition^23,37^. Particularly, the distance-based contact definition employed in this study does not imply chemically relevant contacts between atoms (such as hydrogen bonds or hydrophobic interactions). The chosen cutoff is rather strict and will ignore some meaningful contacts; a relaxation of this cutoff will encompass more contacts but also increases computation time. Our setup explicitly provides secondary structure information during reconstruction which has been shown to improve performance in general^21^ and allows employing this rather strict contact definition as well as ignoring contacts between sequence neighbors (with a sequence separation <6). In consequence, a personal computer can handle the needed computations but no direct comparison to other reconstruction algorithms is possible due to the secondary structure-specific set of used constraints directly depending on CONFOLD^21^. Also, it is natural that CONFOLD^21^, TM-align^46^, and the RMSD as chosen distance measure have an effect on the computed scores and may introduce some form of bias. It would be invaluable to adapt the proposed strategy to other reconstruction algorithms such as Reconstruct^23^ or C2S^25^ and demonstrate the validity of StructureDistiller in a different setup. It is also an open question to what degree the most informative contacts identified in this study are also useful for independent reconstruction algorithms. The TM-score may be more suited to score reconstructs because it is independent of protein length^60^. Another advantage of the TM-score is that it is easy to interpret, especially in the context of deciding whether a reconstruct successfully resembles the fold of the native structure^60,61^. TM-scores are provided as alternative output score for the Structural Relevance. We chose the RMSD value to present results because the majority of readers is familiar with the score and it also provides a direct way to compare results of this study with that of previous publications^23,39,40^. The TM-score correlates well with the RMSD (see Supplementary Fig. 3) and the nature of the findings does not change when the TM-score is considered for analysis. Furthermore, the decision to use 30% of all native contacts to compute the Structural Relevance score is not generally applicable and if more suitable structure-specific values are known they should be used instead. The StructureDistiller algorithm may be improved by determining for each protein structure individually where the sweet spot lies between meaningful reconstructs and maximized sensitivity. Finally, our approach aims at quantifying the information conveyed by a single contact for the integrity of the whole structure. It would be more elegant to express the relevance of a contact using a more rigorously defined, information-theory based approach as described by Nabuurs *et al*.^41^.

In summary, the StructureDistiller algorithm is presented as an approach to assess the structural relevance of individual contacts and residues. This constitutes a novel contribution of the toolkit available for the interpretation of contact maps and protein structures in general, while making the connection of contact maps and tertiary structure more concrete. Maybe the protein folding problem is not solvable without understanding how protein structures can be predicted reliably. In fact, both problems are often described to be two sides of the same coin^16^ and structure prediction did provide new insights into the folding process before^42,43^. Additional tools are needed to make the connection of protein sequence and structure more tangible and StructureDistiller provides just that. The algorithm allows for a novel fine-grained interpretation of contact maps and may improve their interpretability. Applications of the proposed algorithm are not limited to the Start2Fold database^10^, it can be used for the analysis of arbitrary protein structures, e.g. to assess structural effects of mutations at certain residue positions. Following this new paradigm, the interface between protein folding and structure prediction^16^ can be explored in more detail.

## Methods

### Datasets used for evaluation

The Start2Fold database^10^ provides results of pulse labeling hydrogen-deuterium exchange experiments. For the 30 proteins of the dataset (see Supplementary Fig. 1 and supplementary material of^3^ for a detailed definition), 5,173 contacts of 2,529 residues were evaluated. Positions without native contacts were ignored. The Start2Fold database was chosen because it provides a standardized annotation of EFR which initiate the folding process^3,4,44^ and HSR which exhibit significant resilience to unfolding events^10^. This dataset encompasses all major CATH and SCOP classes. Thus, the SR score was assessed using a dataset of proteins for which the folding characteristics are fairly well understood. The size of proteins in the dataset varies from 56–164, which emphasizes relatively small proteins. The covered fold classes are diverse, but present proteins tend to be single domain proteins with fast folding kinetics^44^. Entries without EFR annotation were ignored, even when information on HSR was present. Residues were considered buried when their relative accessible surface area was below 0.16^62^. Evolutionary couplings were computed by the EVfold web server^17,51^. BioJava^63,64^ implementations of the algorithm of Shrake and Rupley^65^ and DSSP^66^ were used for accessible surface area and secondary structure element computation respectively.

### Annotation of residue contacts

A pair of residues was defined to be in contact when the distance between their *C*_*α*_ atoms was less than 8 Å. Contacts maps were created based on this contact definition while ignoring contacts between residues less than six positions apart at sequence level. The remaining tertiary contacts were considered short-range (sequence separation of 6–11), medium-range (12–23), or long-range (>23)^38^. Non-covalent interactions (i.e. hydrogen bonds and hydrophobic interactions) were annotated by PLIP^49^.

### Structure reconstruction and performance scoring

Contact maps (or subsets thereof) were reconstructed as all-atom models by CONFOLD^21^. Secondary structure information of the native structures was annotated by DSSP^66^ and provided as input of the reconstruction routine. By default, CONFOLD creates a set of reconstructs and selects the five top-scoring ones as output. The selected reconstructs and the native structure were then superimposed and their dissimilarity was measured by the RMSD. TM-align^46^ was used for alignment.

### The StructureDistiller algorithm

**Figure 7:**
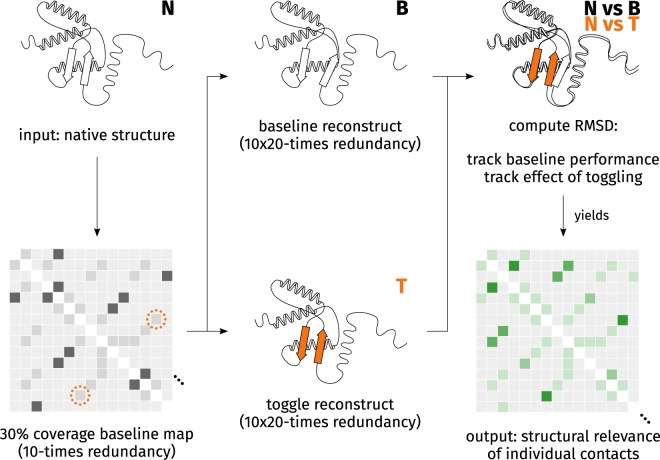
Depiction of the StructureDistiller algorithm. In order to compute the SR score of individual contacts, the effect of their consideration on the reconstruction performance (ΔRMSD) is measured. This allows a novel, more fine-grained interpretation of contact maps. By using 30% of all contacts present in the native structure (N), baseline contact maps are created which provide maximum sensitivity to the removal or addition of a single contact. Baseline reconstructs (B) provide the context to assess the role of individual contacts. Within each baseline contact maps, all contacts of the native contact map are toggled: contacts already present are removed and those absent are added (depicted by orange circle of dots). Reconstructs are created based on these toggle contact maps (T). By superimposing reconstruct and native structure, the SR score of all contacts can be quantified as relative change in RMSD. The idea is that some contacts may provide information which is crucial for reconstruction fidelity, e.g. on the correct arrangement of secondary structure elements (depicted by orange fill).

The StructureDistiller algorithm (Fig. 7) evaluates the structural relevance of individual contacts in the context of a set of other contacts. By selecting 30% of the native contacts of a map, baseline reconstructs can be created which resemble the protein fold and are highly sensitive to the toggling (removal or addition) of an individual contact. The performance of the baseline reconstructs can be quantified by a structural alignment to the native structure. Analogously, the performance can be measured for the toggle reconstructs, which represent the information conveyed by one particular contact. By comparing the performance of a toggle reconstruct with its corresponding baseline reconstruct, the SR score of all contacts is quantified.

The StructureDistiller algorithm is presented in Algorithm 1. A protein structure *S*_native_ in legacy PDB format is the input. Structure files should encompass single domains of a single chain. The corresponding contact map *C*_native_ is created. *C*_native_ constitutes the set of all contacts which will be evaluated.

Fractions equal to 30% of *C*_native_ are then randomly selected. The SR score of a contact depends on all other contacts used for a reconstruction. No effect can be expected when a contact is considered which contributes no additional, but only redundant information^40^. The creation of random subsets of *C*_native_ is performed with a redundancy *r* of 10. The resulting subset of contacts ${C}_{{\rm{baseline}},i}$ is used to create the baseline reconstructs ${S}_{{\rm{baseline}},i}$. The average ${{\rm{RMSD}}}_{{\rm{baseline}},i}$ of each created subset ${C}_{{\rm{baseline}},i}$ is tracked with respect to *S*_native_. These subsets are highly sensitive to the removal and addition of a single contact and the basis for all further computations.

All contacts of *C*_native_ are now evaluated regarding their SR by pairing each contact to each baseline subset of contacts ${C}_{{\rm{baseline}},i}$. For each pair, it is determined whether the current contact *c* is element of ${C}_{{\rm{baseline}},i}$. If so, *c* is removed from ${C}_{{\rm{baseline}},i}$, else *c* is added to the corresponding subset. The change in reconstruction performance can be quantified by this toggling of a contact: the modified subset ${C}_{{\rm{toggle}},i}$ is again used for a reconstruction and ${{\rm{RMSD}}}_{{\rm{toggle}},i}$ is used to describe its quality. The average improvement of the reconstruction with knowledge of the contact *c* is tracked by Δ${{\rm{RMSD}}}_{c}$. ${{\rm{RMSD}}}_{{\rm{baseline}},i}-{{\rm{RMSD}}}_{{\rm{toggle}},i}$ is evaluated when *c* was added to the subset, the expression is flipped when *c* was removed. The SR of individual residues is the average of all Δ${{\rm{RMSD}}}_{c}$ of contacts this residue participates in. Positive SR scores represent contacts which increase reconstruction fidelity while negative scores occur for contacts hindering reconstruction. The influence of individual residues can be computed by summing up the SR scores of its contacts.

The runtime of StructureDistiller scales with the number of contacts in the initially created map *C*_native_. The individual reconstruction tasks are distributed among worker threads which allows for efficient parallelization. Using a conventional workstation, computation on proteins with up to 200 residues requires one day on average.

### Definition of reconstruction strategies

Various subset selection strategies were used to assess the relevance of contacts in a contact map. In all cases, a number equal to 30% of the contact count in the native map was used. For the creation of the random bin, 30% of all native contacts were chosen randomly. The most relevant selection constitutes the 30% of all contacts sorted for highest SR, least relevant resembles 30% of all contacts with the lowest scores. All percentage numbers are relative to the number of contacts in the native structure. All operations on all definitions are performed with ten-fold redundancy. Contact distances were assessed: all short-range (sequence separation of 6–11) and long-range (>23) contacts^38^ were assessed. The same was done for hydrogen bonds and hydrophobic interactions. Because the number of contacts of a particular distance or type may be smaller than 30%, a dedicated bin (e.g. non-short) was created to match in size.

**Algorithm 1:**
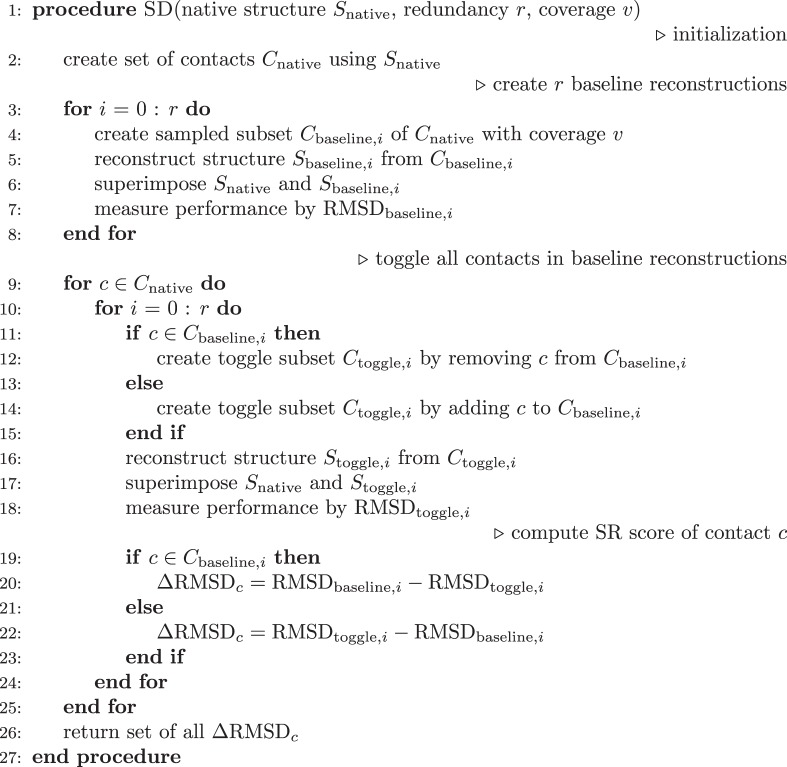
StructureDistiller Pseudocode.

### Introduction of non-native contacts

Non-native contacts are contacts not present in the contact map of the native protein structure. Contact maps were created by the best and random strategy and in 1% bins up to 10% non-native contacts were introduced, replacing the initially selected native contacts. Analogous to the employed contact definition, non-native contacts were required to exhibit a sequence separation greater than five.

### Statistical analysis

Residues without any contacts (i.e. where no SR score can be computed) were ignored from statistical analysis. Notched box plots were used for visualization. The notch corresponds to the 95% confidence interval around the median. When the notches of two distributions do not overlap, they can be assumed to be different. Significance was explicitly tested by a two-tailed Mann-Whitney U test. *p*-values < 0.05 were considered significant.

## Supplementary information

Supplementary information

## Data Availability

A reference implementation of the StructureDistiller algorithm is available in the module structural-information at https://github.com/JonStargaryen/jstructure. A compiled version is deposited at 10.5281/zenodo.1405369. All evaluated data is included in the manuscript and its Supplementary Information^67^.
