# Supplementary material for: StructureDistiller: Structural relevance scoring identifies the most informative entries of a contact map
Source: Sci Rep. 2019 Dec 6;9:18517. doi: 10.1038/s41598-019-55047-4 (PMC6898053; doi:10.1038/s41598-019-55047-4)
Supplement: Supplementary file 1 — Supplementary information [file 41598_2019_55047_MOESM1_ESM.pdf]

StructureDistiller: Structural relevance scoring identifies the most informative entries of a contact map

Sebastian Bittrich, Michael Schroeder, Dirk Labudde

Supplementary Figure 1

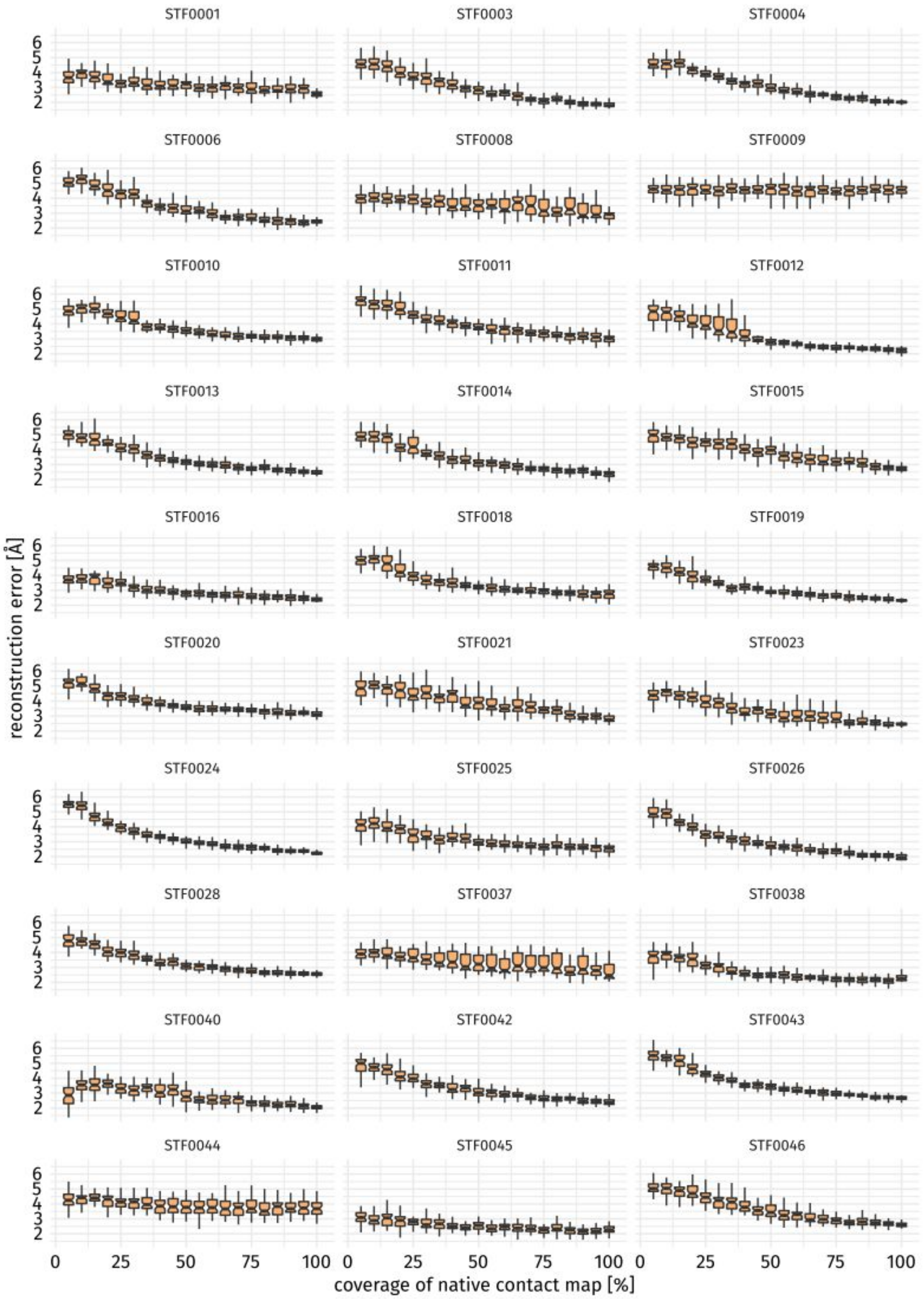

Reconstruction error by percentage of contacts split by protein.
For some proteins the reconstruction performance does not increase when more or even all native contacts are considered. In other cases, reconstructions with a large number of constraints exhibit high variance regarding their reconstruction performance.

Supplementary Figure 2

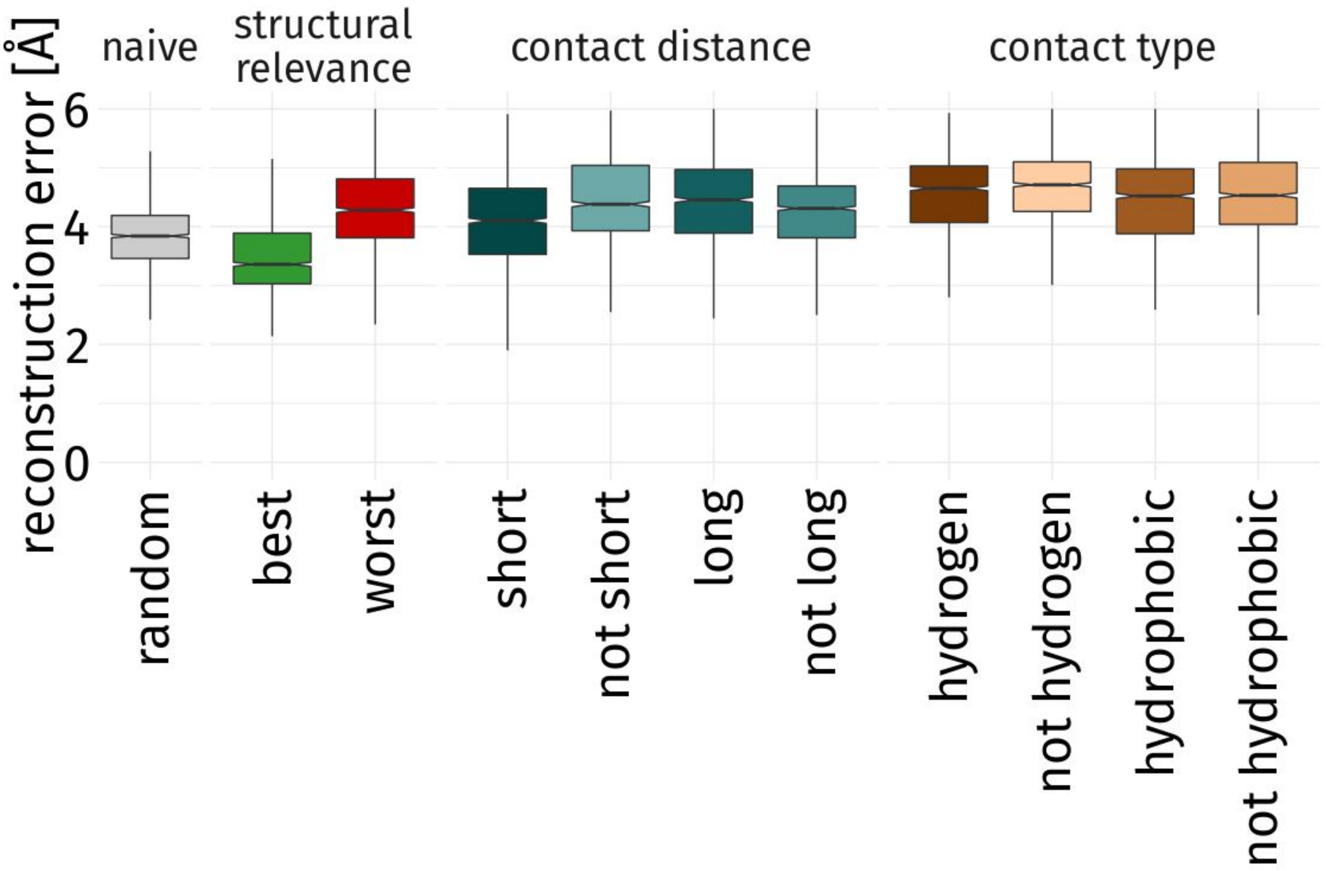

Detailed impact on reconstruction performance by strategy.
Various strategies were used to reconstruct structures of the dataset using a number of constraints equal to 30% of contacts in the native map. Contact distance and type bins are only comparable to the explicitly negated bins because the available number of contacts differs (i.e. there may not be enough hydrogen bonds to match the number of contacts in the random bin). 'Not' selections based on contact distance or type perform worse or on par than their counterpart which implies the necessity to consider a complex collection of contacts for a successful reconstruction (see Chen et al., 2007). When combined short contacts yield relatively good reconstructs even though their structural relevance scores are low. All contact distance and type bins differ significantly from their respective counterpart.

Supplementary Figure 3

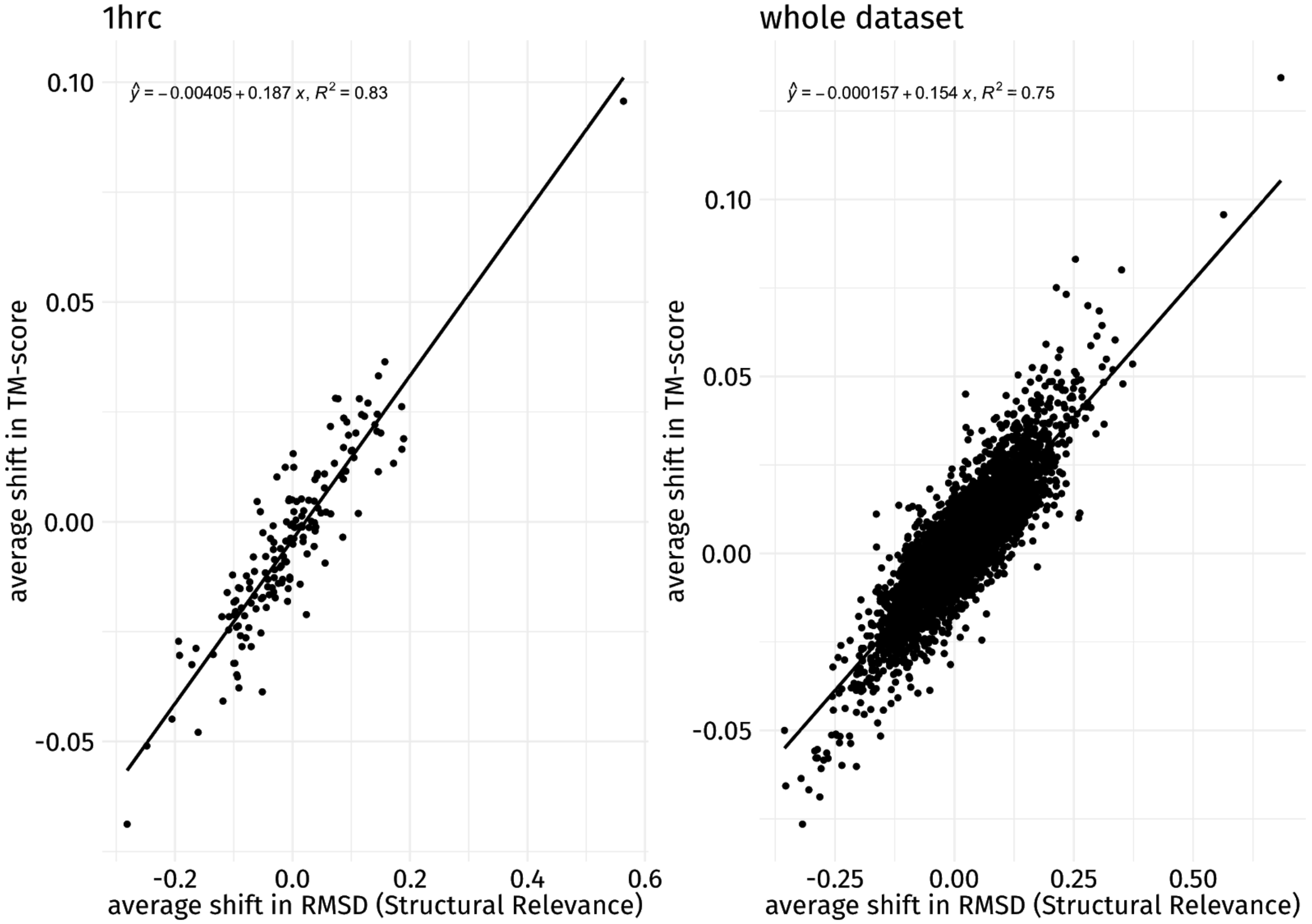

Impact of the choice of similarity/dissimilarity measure.

There is no consensus on the best measure for structure similarity or dissimilarity. The TM-score (see Zhang & Skolnick, 2004) addresses issues such as the size dependency of the RMSD and is commonly used to score global structure similarity. StructureDistiller reports both RMSD and TM-score. This figure shows a strong correlation between RMSD and TM-score for both individual proteins as well as the complete dataset. As the nature of the results does not change when considering the TM-score, we choose the RMSD to present results because more readers are familiar with it.
